# 2‐Amino‐3*H* ‐phenoxazin‐3‐one is a lead inhibitor of *Cuscuta campestris* early development: structure–activity relationships of *o* ‐aminophenol‐derived allelochemicals

**DOI:** 10.1002/ps.71117

**Published:** 2026-07-12

**Authors:** Cristina Díaz‐Franco, Mónica Fernández‐Aparicio, José MG Molinillo, Rosa M Varela, Francisco A Macías, Jesús G Zorrilla

**Affiliations:** ^1^ Allelopathy Group, Department of Organic Chemistry Facultad de Ciencias, Institute of Biomolecules, University of Cadiz Puerto Real Spain; ^2^ Department of Crop Protection Institute for Sustainable Agriculture, CSIC Córdoba Spain

**Keywords:** agrochemistry, phenoxazinones, allelochemicals, parasitic weeds, pest management, structure–activity relationships

## Abstract

**BACKGROUND:**

2‐Amino‐3*H*‐phenoxazin‐3‐one (synonym: questiomycin A; abbreviated as APO or Phx‐3) is a naturally occurring phenoxazinone‐type allelochemical with recognized potential as drug scaffold, but its value against parasitic weeds remains unexplored. Dodders, represented by species like *Cuscuta campestris*, are destructive parasitic plants that compromise crop productivity and quality, as well as damage to ornamental plants. Control options are limited and often rely on herbicides with sustainability and selectivity constraints.

**RESULTS:**

Inspired by allelopathy as a source of eco‐compatible herbicide models, APO and a set of phenoxazinones obtained via one‐step oxidative cyclocondensation of aminophenols (6–91% yields) were evaluated on *C. campestris* germination and early seedling development at 100–400 μM. APO, synthesized in 70% yield from 2‐aminophenol, showed the most consistent and specific inhibitory activity on both germination and seedling growth. In addition, APO induced marked necrotic effects, which were further enhanced by its nitro‐derivative, causing around 90% necrosis across the tested concentration range. Some additional derivatives displayed moderate necrosis levels, enabling preliminary structure–activity relationship insights within the phenoxazinone scaffold, suggesting a trend in favor of lower‐cLog *P* structures that share similar topological polar surface area values.

**CONCLUSIONS:**

This is the first evidence supporting phenoxazinones, particularly the allelochemical APO, as leads for parasitic weed and dodder management. Their synthetic accessibility at multigram scale from aminophenols, including by‐products and contaminants from industrial wastewaters like 2‐aminophenol, strengthens a real applicability from a circular‐chemistry perspective for development of active agents based on natural products in integrated control strategies for a more sustainable pest management. © 2026 The Author(s). *Pest Management Science* published by John Wiley & Sons Ltd on behalf of Society of Chemical Industry.

## Graphical abstract

Phenoxazinones caused differential types of phytotoxic effects in *C. campestris* early development. APO is a coherent lead inhibiting *C. campestris* germination and early seedling growth.

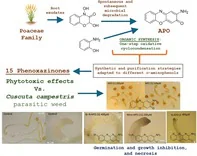

## 1 INTRODUCTION

Weeds represent a persistent and evolving threat to global agriculture, causing significant losses in crop quality and productivity. Globally, it is estimated that weeds can cause an average loss of 10–30% of agricultural production, although in severe infestations or in specific crops under severe infestation or in particularly susceptible crops, yield losses may become substantially higher and, in extreme cases, lead to complete crop failure.^1^ In the current context of climate change, reduced crop resilience, increasing restrictions on herbicide use, and the global demand for sustainable intensification of food production, weed pressure is expected to intensify further, amplifying their economic and agronomic impact if proper management strategies are not employed.^2^ Within this context, parasitic weeds, such as *Cuscuta campestris* (commonly known as field dodder), represent a particularly problematic weed due to their unique life cycle and mode of interaction with host plants. Unlike autotrophic weeds, which primarily compete for soil resources and light, *C. campestris* establishes a direct connection with host vascular system (Fig. 1), extracting water and nutrients through specialized invasive structures known as haustoria that attach to host aerial tissues.^3^ This intimate association, characterized by physical connection and developmental synchronization, leads to drastic reductions in crop yield, and also hinders effective control as they limit the applicability and efficacy of conventional weed management strategies without causing damage to the host crop. Reports include cases where uncontrolled infestation of alfalfa (*Medicago sativa*) with *C. campestris* caused forage yield reductions by 73% in 2 years of field experiments, and tomato yield losses as high as 75% under dodder pressure.^4^, ^5^ The difficulty in controlling *C. campestris* is exacerbated by its wide host range, which includes a variety of herbaceous and woody agricultural crops, such as alfalfa, buckwheat (Fig. 1), clovers, sugar beet, tomato, tobacco, and other vegetables, including onion and potato, and weeds.^6^, ^7^, ^8^, ^9^ Its dispersal mechanisms are highly efficient, encompassing wind, water, animals, and contaminated planting material, and the ability to spread from plant to plant or re‐establish from plant fragments, all this further complicating physical control methods. Thus, the parasitic nature of *C. campestris*, and particularly its dependence on an immediate connection with a host after germination for survival, necessitates control strategies that extend beyond simple germination inhibition. For this specific weed, assessing seedling growth inhibition is fundamental for determining practical herbicidal potential, as it directly targets the early establishment and host‐attachment stages that are essential for successful parasitism and subsequent weed survival.

**Figure 1:**
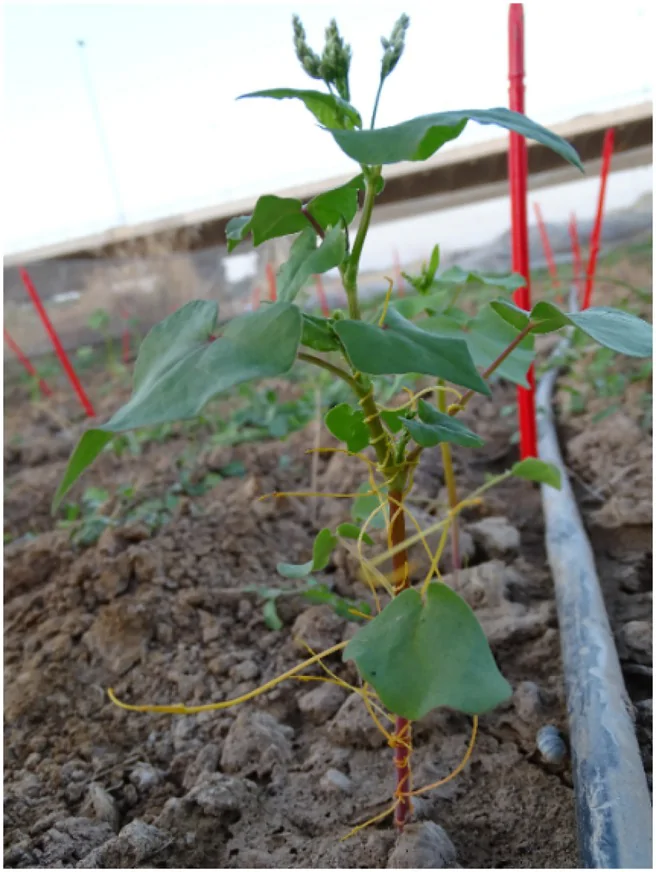
Buckwheat (*Fagopyrum esculentum*) plant parasitized by *Cuscuta campestris*.

Despite initial efficacy of classical herbicides, their widespread and continuous application has generated significant challenges. These include the persistence of herbicide residues in soil, which can alter soil microbial communities and soil functions (including nutrient cycling and enzyme activities) and may also contribute to groundwater contamination through leaching.^10^, ^11^ Moreover, the inherent lack of specificity of many pesticides results in harm to non‐target organisms and their incorporation into the food chain. For example, soil‐applied neonicotinoids (imidacloprid and dinotefuran) have been detected in nectar of treated urban plants at concentrations reported to exceed levels associated with adverse effects on bees, while pesticide residues such as DDT‐related compounds can remain bioavailable and biomagnify along terrestrial agroecosystem food chains.^12^, ^13^ An additional critical aspect, related to effectiveness, is that the continued use of agents with recurrent modes of action has led to a widespread resistance concern, with weeds having developed resistance to 21 of the 31 known herbicide modes of action.^14^ In this context, natural products, especially those derived from allelochemicals, represent a viable and more environmentally friendly alternative for the development of innovative bioherbicides.

Phenoxazinones, with special emphasis on the aminophenoxazinones, are a class of secondary metabolites derived from the natural degradation of benzoxazinones. These parent compounds are exuded by the roots of cereal crops in the Poaceae family, such as maize, wheat, and rye. Once in the soil, they get degraded by microorganisms and are converted into bioactive phenoxazinones like 2‐amino‐3*H*‐phenoxazin‐3‐one (also known as questiomycin A, and commonly abbreviated as APO or Phx‐3).^15^, ^16^ Within phenoxazinones, APO (**1**) stands out for its potent phytotoxic activity, having demonstrated relevant percentages of seedling growth inhibition in previous studies with non‐parasitic weeds like *Lolium rigidum*, *Plantago lanceolata* or *Portulaca oleracea*.^17^, ^18^ A particularly relevant mechanistic feature of APO (**1**) is its reported inhibition of histone deacetylase activity, which points to specific epigenetic interference as a basis for phytotoxicity rather than only nonspecific toxicity.^19^ Because plant histone deacetylases regulate chromatin acetylation and transcriptional programs that control seed germination, seedling development, and stress responses,^20^, ^21^ that bioactivity of APO (**1**) gives this scaffold special interest as a bioherbicide lead capable of perturbing developmental pathways in competing plants.

The practical use of phenoxazinones is also attractive because they could be obtained via organic synthesis following oxidative cyclocondensation of readily available aminophenols, and gram‐scale preparations of APO (**1**) and other phenoxazinones are feasible.^22^, ^23^, ^24^ This synthetic accessibility is relevant for agrochemical development and gains additional interest from a circular‐chemistry perspective, since 2‐aminophenol, the key scaffold for APO (**1**) preparation, is a documented contaminant in some industrial wastewaters that require dedicated treatment.^22^, ^25^ In this context, recovery of 2‐aminophenol from such streams could be viewed as a valorization strategy to generate phenoxazinone scaffolds for agrochemical applications from a circular‐chemistry perspective, rather than relying exclusively on waste removal.

Based on the known phytotoxicity of APOs and the pressing need for novel dodder control agents, this research aimed to synthesize a library of APO derivatives with targeted modifications to change physicochemical properties and biological activity. This study specifically evaluates the phytotoxic potential of these novel compounds against *C. campestris*, the first time on a parasitic weed, by measuring effects not only in seed germination and growth, but also in relation to necrotic effects. Previous bioprospecting studies on bioactive scaffolds against *C. campestris* have highlighted mainly simple aromatic metabolites,^26^, ^27^ supporting further exploration of more complex and potentially more selective allelochemical scaffolds. This article highlights the potential of phenoxazines for effective *C. campestris* control and offers an in‐depth exploration of structure–activity relationships.

## 2 MATERIALS AND METHODS

### 2.1 Synthesis, purification, and structural characterization of APO and its derivatives

This study focused on the molecules of APO (**1**) and the structurally‐related phenoxazinones 2‐amino‐3*H*‐dipyrido[3,2‐b:2′,3′‐e][1,4]oxazin‐3‐one (**2**), 2‐amino‐8‐bromo‐3*H*‐benzo[b]pyrido[2,3‐e][1,4]oxazin‐3‐one (**3**), 2‐amino‐7‐bromo‐3*H*‐dipyrido[3,2‐b:2′,3′‐e][1,4]oxazin‐3‐one (**4**), 2‐amino‐8‐bromo‐3*H*‐phenoxazin‐3‐one (**5**), 2‐amino‐4,8‐dibromo‐1*H*‐phenoxazin‐1‐one (**6**), 2‐amino‐8‐chloro‐3*H*‐phenoxazin‐3‐one (**7**), 2‐amino‐8‐chloro‐4‐iodo‐3*H*‐phenoxazin‐3‐one (**8**), 2‐amino‐8‐fluoro‐3*H*‐phenoxazin‐3‐one (**9**), 2‐amino‐8‐fluoro‐4‐iodo‐3*H*‐phenoxazin‐3‐one (**10**), 2‐amino‐8‐nitro‐3*H*‐phenoxazin‐3‐one (**11**), 2‐amino‐3‐oxo‐3*H*‐phenoxazine‐8‐carboxilyc acid (**12**), 2‐amino‐8‐amino‐3*H*‐phenoxazin‐3‐one (**13**), 2‐amino‐3‐oxo‐3*H*‐phenoxazine‐7‐carboxilyc acid (**14**), and 2‐amino‐4‐iodo‐3‐oxo‐3*H*‐phenoxazine‐7‐carboxilyc acid (**15**) (Fig. 2).

**Figure 2:**
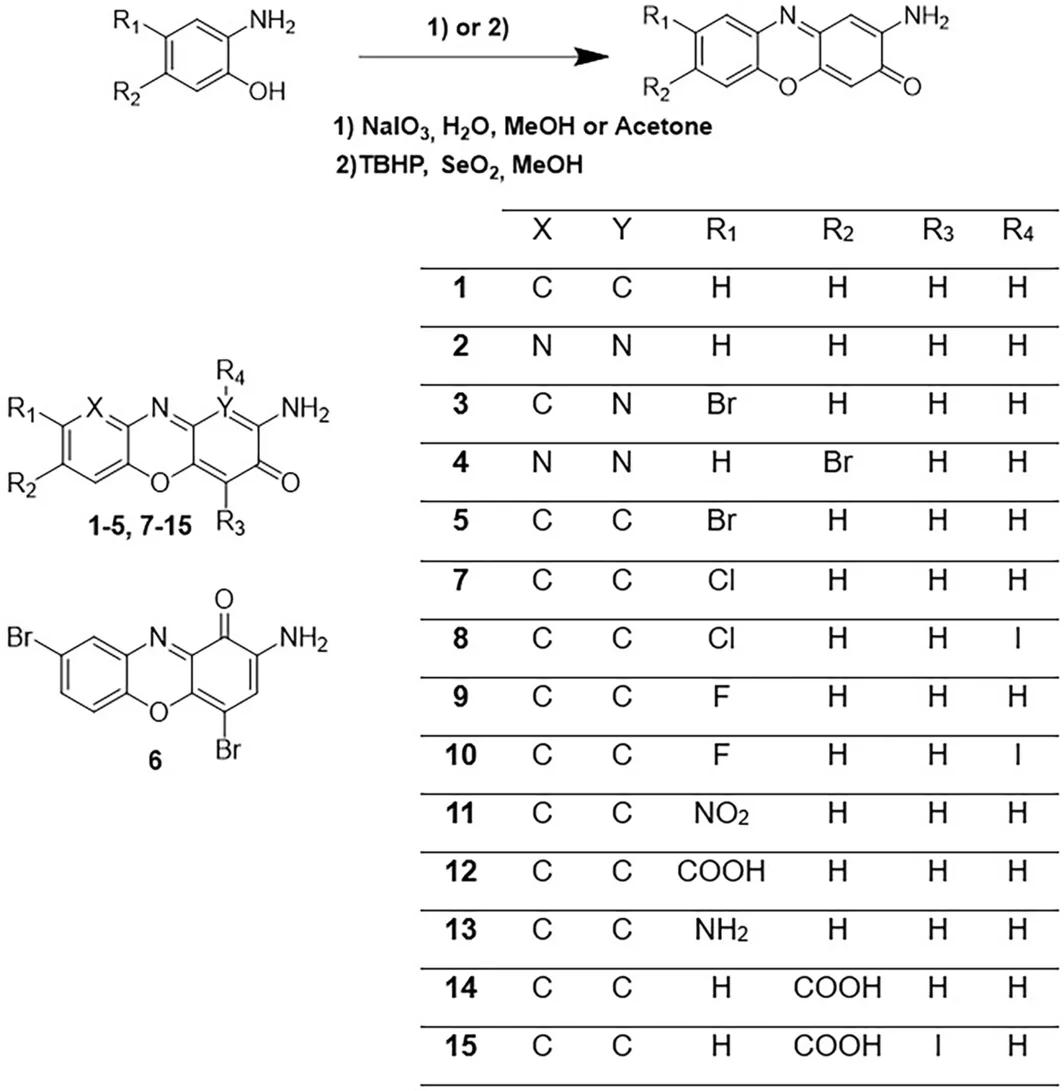
Structures of APO (**1**) and the phenoxazine derivatives **2–15**.

APO (**1**) and the phenoxazinones **2–12**, **14** and **15** were synthesized via oxidative cyclocondensation of *o*‐aminophenols following two distinct approaches. These two different strategies were used because the NaIO_3_‐mediated synthesis efficiently afforded key phenoxazinones in good yield like APO (**1**), but can promote iodinated byproducts with several substituted *o*‐aminophenols, whereas *tert*‐butyl hydroperoxide (TBHP)‐mediated oxidation avoided iodination in compatible substrates, although it was not suitable for more oxidation‐sensitive acidic phenols. Notably, in some of the reactions involving two different *o*‐aminophenols, minor products tentatively identified as byproducts from competing homo‐ and heterocondensation pathways were observed (mainly in the syntheses of products **3** and **11**).

The first approach used NaIO_3_ as oxidant, generating the intermediates that undergo oxidative coupling to afford the phenoxazine core after intramolecular ring closure. An aqueous solution of NaIO_3_ was prepared, and a solution of the corresponding aminophenol in methanol or acetone was added dropwise under stirring and maintained for 10 min. A second portion of the same or a different *o*‐aminophenol dissolved in methanol or acetone was then added dropwise, and the mixture was stirred overnight at room temperature. The crude reaction mixture was cooled in an ice bath (20 min), the precipitate was collected by vacuum filtration, and washed with cold water to remove inorganic salts and iodine species. The resulting solid contained the desired phenoxazinone (**1**, **2**, **4**, **5**, **7**, **8**, **10–12**, **14** or **15**).

The second approach used TBHP as the terminal oxidant, in the presence of SeO_2_ as redox mediator catalyst to promote oxidation of the aminophenols enabling oxidative C–N or C–O couplings and subsequent cyclization to the phenoxazinone scaffold. In some substrates, these oxidative conditions appeared to shift the reaction pathway from the conventional APO‐type kinetic cyclization toward alternative regioisomeric products, consistent with a substrate‐dependent balance between kinetic and thermodynamic oxidative coupling modes. SeO_2_ (0.5 equiv) was added to a solution of the *o*‐aminophenol substrate in methanol, followed by TBHP (2.0 equiv) added dropwise via glass syringe. The reaction mixture was stirred at room temperature for 1 h. The heterogeneous SeO_2_ was removed by filtration, and residual TBHP was removed under reduced pressure to afford the crude product for further purification of the phenoxazinone (**3**, **6** and **9**).

Product **13** was obtained by reduction of compound **11**, which was first dissolved in acetone, then a 0.3 M solution of NH_4_Cl and zinc powder were added. The reaction mixture was sonicated for 1 h, then filtered through Celite to remove Zn and extracted with *n*‐butanol to remove NH_4_Cl.^28^

Products **1**, **2**, **4**, **6** and **15** did not require further purification steps after work‐up of the reaction. Purification of products **3**, **5** and **7–13** was performed by centrifugal partition chromatography (CPC) following the methodology detailed elsewhere^23^ in a Gilson CPC system (Saint Avé, France) paired with a Gilson PLC 2250 unit comprising a binary pump, a UV detector, and a fraction collector. Three CPC devices were employed depending on the rotor capacity: fast‐CPC (FCPC; 50 mL), mid‐scale‐CPC (mCPC; 250 mL), and conventionalCPC (cCPC; 100 mL). Experiments were conducted in either ascending or descending mode, determined by the phase designated as stationary. Ascending mode was used when the denser phase served as the stationary phase, whereas descending mode was applied when the denser phase acted as the mobile phase. The choice of mode depends on the partition coefficients of the components in the mixture. Solvent systems, consisting of *n‐*hexane/ethyl acetate/methanol/1,2‐dimethoxyethane/water mixtures, were selected based on the partition coefficients determined using the shake flask method. Product **11** was purified by column chromatography with a *n‐*hexane/ethyl acetate mixture (6:4 v/v) and it was also obtained pure in the CPC purification of crude mixture of product **13**. Product **14** was purified by HPLC in a VARIAN ProStar, model 210 using a semi‐preparative column Gemini 5 μm (110 Å, 250 × 10 mm) and a diode‐array (DAD) as a detector, selecting the wavelengths of 290 and 420 nm. The mobile phase of water +0.1% acetic acid (solvent A) and acetonitrile +0.1% acetic acid (solvent B) at a 3 mL min^−1^ flux followed the optimized gradient system 0–5 min at 30% B, 5–25 min in a linear increment to 100% B, and 25–30 min in a linear increment to 30% B.

Procedures for the synthesis of each product and their purification are summarized in Table 1. Structural elucidation and purity assessment of all products were determined by high‐resolution mass spectrometry (HRMS) following positive or negative ionization mode, and by comparison of the experimental NMR spectra (provided as Supporting Information) with spectroscopic data available in literature.^17^, ^23^ ^1^H and ^13^C NMR spectra were recorded at room temperature using deuterated dimethyl sulfoxide (DMSO‐*d*_*6*_) as the solvent on a Bruker spectrometer at 500/125 MHz (Karlsruhe, Germany). The resonance of residual DMSO‐*d*_*6*_ was set to δ 2.50 ppm for ^1^H and 39.5 ppm for ^13^C. Exact masses were measured on a Waters time‐of‐flight mass spectrometer (XEVO‐G2S‐QTOF) with electrospray ionization (Milford, MA, USA) located at the Institute of Biomolecules (INBIO). All reagents were purchased from Aldrich Chemical Co. (St. Louis, MO, USA), and solvents from VWR International (Radnor, PA, USA).

**Table 1:** Strategies employed for the synthesis of APO (**1**) and its derivatives **2–15**

Product	Starting aminophenol (g, mmol)	Reagents and solvent	Time	Yield (%)	CPC strategy
**1**	2‐Aminophenol (0.250 g, 2.3 mM)	NaIO_3_/acetone (0.430 g, 2.2 mM)	24 h	70	‐
**2**	2‐Aminopyridin‐3‐ol (0.330 g, 3 mM)	NaIO_3_/MeOH (0.337 g, 1.7 mM) (0.722 g, 3.6 mM)	24 h	81	‐
**3**	2‐Aminopyridin‐3‐ol (0.200 g,1.8 mM), and 2‐amino‐4‐bromophenol (0.345 g, 1.8 mM)	TBHP + SeO_2_/MeOH	24 h	18	FCPC, HEMWat 0 (*n‐*hexane/ethyl acetate/methanol/water; 1/1/1/1), 1 mL min^−1^ at 800 rpm, asc
**4**	2‐Amino‐6‐bromopyridin‐3‐ol (0.124 g, 0.7 mM)	NaIO_3_ (0.174 g, 1 mM/MeOH)	24 h	50	‐
**5**	2‐Amino‐4‐bromophenol (0.140 g, 0.7 mM)	NaIO_3_ (0.197 g, 1 mM/MeOH)	24 h	40	FCPC, HEMDmeWat +2 (*n*‐hexane/ethyl acetate/methanol/1,2‐dimethoxy ethane/water; 2/1/1/1/1), 0.7 mL min^−1^ at 800 rpm, asc
**6**	2‐Amino‐4‐bromophenol (0.282 g, 1.5 mM)	TBHP + SeO_2_/MeOH	1 h	89	‐
**7**	2‐Amino‐4‐chlorophenol	NaIO_3_/MeOH	24 h	30	mCPC, HEMDmeWat 0 (*n‐*hexane/ethyl acetate/methanol/1,2‐dimethoxy ethane/water; 1/1/1/1/1), 2.5 mL min^−1^ at 2000 rpm, asc
**8**				40	
**9**	2‐Amino‐4‐fluorophenol (0.193 g, 1.5 mM)	TBHP + SeO_2_/MeOH	1 h	51	FCPC, HEMDmeWat 0 (*n*‐hexane/ethyl acetate/methanol/1,2‐ dimethoxy ethane/water; 1/1/1/1/1), 1 mL min^−1^ at 800 rpm, asc
**10**	2‐Amino‐4‐fluorophenol (0.100 g, 0.8 mM)	NaIO_3_/MeOH (0.318 g, 1.6 mM)	24 h	91	
**11**	2‐Aminophenol (0.280 g, 1.8 mM) and 2‐amino‐4‐nitrophenol (0.480 g, 3 mM)	NaIO_3_/acetone (0.980 g, 5 mM)	24 h	25	cCPC; ARIZONA L (*n*‐hexane/ethyl acetate/methanol/water; 2/3/2/3),5 mL min^−1^ at 1200 rpm, dsc
**13**	Product **11** (0.450 g 1.7 mM)	Zn (0.8 g, 12 mM) + NH_4_Cl (0.8 g, 15 mM)/acetone	2 h	55	
**12**	3‐Amino‐4‐hydroxybenzoic acid (0.153 g,1 mM)	NaIO_3_/MeOH (0.110 g, 0.5 mM) (0.237 g, 1.2 mM)	24 h	6	FCPC; HEMDmeWat 0 + 0.1% FA (*n*‐hexane/ethyl acetate/methanol/1,2‐ dimethoxy ethane/water; 1/1/1/1/1), 1 mL min^−1^ at 800 rpm, dsc
**14**	4‐Amino‐3‐hydroxybenzoic acid (0.153 g, 1 mM)	NaIO_3_/MeOH (0.110 g, 0.5 mM) (0.237 g, 1.2 mM)	24 h	84	–
**15**				11	

Strategies for the purification of products **3**, **5** and **7–13** by centrifugal partition chromatography; asc: ascending mode, dsc: descending mode; FCPC (50 mL), mCPC (250 mL), and cCPC (100 mL).

### 2.2 Bioassays with *Cuscuta campestris*

Bioassays were performed as described in established protocols,^26^ allowing a comparative early‐development screening under standardized conditions. To break the physical dormancy imposed by the thick seed coat of *C. campestris* (allowing staggered germination and persistence of the soil seed bank),^27^ seeds were chemically scarified by immersion in concentrated H_2_SO_4_ for 45 min. Seeds were then thoroughly rinsed with distilled water until neutral and air‐dried in a laminar‐flow cabinet. Scarified seeds (*n* = 10 per dish) were placed with sterile tweezers onto sterilized filter paper discs (5.0 cm diameter) positioned in Petri dishes (5.5 cm diameter). Test phenoxazinones were prepared from 1.0 mM stock solutions initially dissolved in 2% dimethyl sulfoxide (DMSO) in sterile distilled water, and subsequently diluted with sterile distilled water to final concentrations of 400, 200, and 100 μM (final solvent content kept constant across treatments). Triplicate 1.0‐mL aliquots of each treatment were applied to the filter paper discs. Samples containing just sterile distilled water with 2% DMSO were used as controls, since this solvent concentration does not trigger phytotoxic effects on *C. campestris* seedlings, therefore confirming that phytotoxic effects in phenoxazinone‐treated samples were not caused by the solvent vehicle. Petri dishes were sealed with Parafilm, wrapped in aluminum foil, and incubated in the dark in a growth chamber at 23 ± 1 °C and 65% relative humidity for 5 days.

Seedling growth for each treatment was calculated relative to the seedling growth of the corresponding control, while photographs were taken of root apices when seedlings had developed necrosis. The percentage of seedlings that developed a necrotic root was calculated in each triplicated disk for each treatment. Seedlings were observed using a stereoscopic microscope (Leica S9i; Leica Microsystems GmbH, Wetzlar, Germany). All bioassays were performed using a completely randomized design. Percentage data were approximated to normal frequency distribution by means of angular transformation, with percentage data subjected to analysis of variance (ANOVA). The significance of mean differences among treatments was evaluated by Tukey at *P* < 0.05. Statistical analysis was performed using SPSS software 27 (SPSS Inc., Chicago, IL, USA).

Calculation of CLogP and topological polar surface area (TPSA) values was performed using, respectively, by ChemOffice v20.1 (PerkinElmer, Waltham, MA, USA) and SwissADME software.^28^, ^29^, ^30^

## 3 RESULTS AND DISCUSSION

APO (**1**) and its structural derivatives **2–15** exhibited differential phytotoxic effects in *C. campestris* seedlings (Fig. 3), with a marked structure‐dependent response and a clear specificity for APO (**1**) in relation to inhibition of both germination and early seedling growth. Considering these effects, none of the derivatives **2–15** achieved a significant potency like APO (**1**), which showed inhibitions of 88.6 ± 3.7% in germination and 89.0 ± 3.5% in growth at 400 μM. At the lowest test concentration – quartering the highest concentration to 100 μM – these values just decreased to 47.4 ± 4.9 and 64.7 ± 2.0%, respectively, being the most potent metabolite on *C. campestris* early development reported in the literature to the best of our knowledge.^26^ The estimated IC_50_ values of APO (**1**) were 91.1 and 55.9, respectively, for inhibition of germination and seedling growth. These values should be considered apparent estimates derived from extrapolation below the tested concentration range.

**Figure 3:**
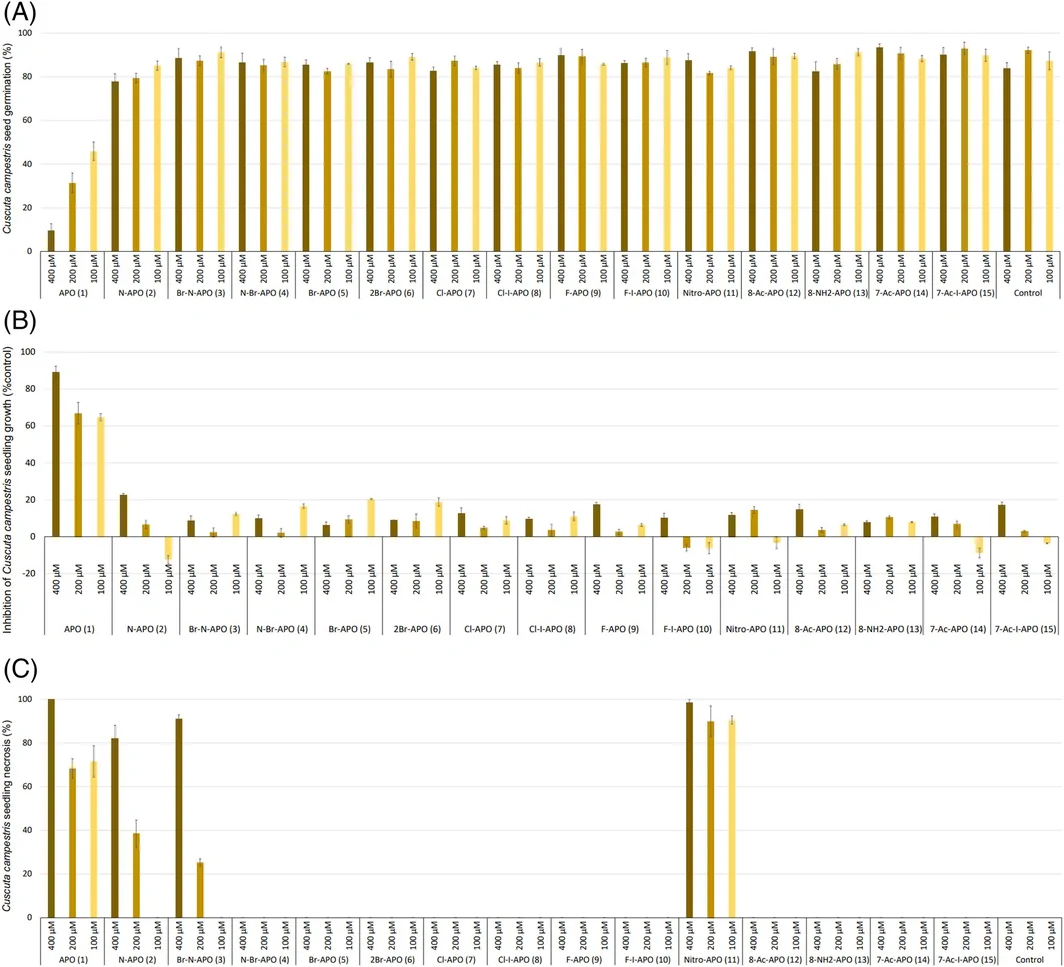
*In vitro* assessment of *Cuscuta campestris* germination inhibition (A), seedling growth inhibition (B), and percentage of necrosis (C) induced by treatment with products **1–15**.

This selectivity supports the view that APO (**1**) is a privileged allelochemical scaffold targeting biological processes in *C. campestris* germination or growth. Importantly, APO (**1**) itself is not merely a synthetic lead, being a naturally occurring phenoxazinone that accumulates in agroecosystems as a stable, bioactive end‐product of benzoxazinoid degradation or biotransformation, consistent with a key ecological role in allelopathy and plant–plant interference.

When necrotic‐like responses were assessed in *C. campestris* seedlings (Fig. 4), APO (**1**) showed strong activity, reaching 100% of seedlings with necrotic symptoms at 400 μM and remaining close to 70% at the lowest tested doses (Figs 3(C) and 4). In contrast to the patterns observed for germination and growth inhibition, several derivatives displayed measurable necrotic effects. The most pronounced necrosis‐inducing activity was observed for the nitro‐phenoxazinone (**11**), which increased necrosis compared to APO (**1**) at concentrations of 200 and 100 μM (Fig. 3(C)). This trend is consistent with previous work reporting that nitration of the APO scaffold can enhance phytotoxicity in a species‐dependent manner, including improved inhibition of *P. oleracea* at low concentration.^17^ Phytotoxicity enhancement after introducing a nitro group has also been documented in other phenolic structures, serving as an example the case of 4‐nitroguaiacol, which was more phytotoxic than its non‐nitrated analogue guaiacol in experiments with sunflower, with effects suggested to involve perturbation of lignin biosynthesis after uptake.^31^ Across plant systems, previous work has reported phytotoxic responses to nitroaromatic structures, offering a helpful structure–activity framework to contextualize the enhanced necrotic profile observed here for the APO scaffold.^32^, ^33^ A complementary explanation may involve redox imbalance, since nitroaromatic compounds can undergo enzymatic reduction to reactive intermediates and participate in redox‐cycling processes, which may promote reactive oxygen species (ROS) formation.^34^, ^35^ Consistently, nitrobenzene has been reported to inhibit seedling growth and induce oxidative stress in rice, while excessive ROS accumulation in plant tissues is widely associated with lipid, protein, and nucleic‐acid oxidation and, under severe stress, cell death.^35^, ^36^ Therefore, the stronger necrosis observed for the nitro‐substituted phenoxazinone (**11**) may reflect not only changes in uptake or lignification‐related processes, but also an increased capacity to disturb redox homeostasis in the highly sensitive tissues of pre‐attached *C. campestris* seedlings.

**Figure 4:**
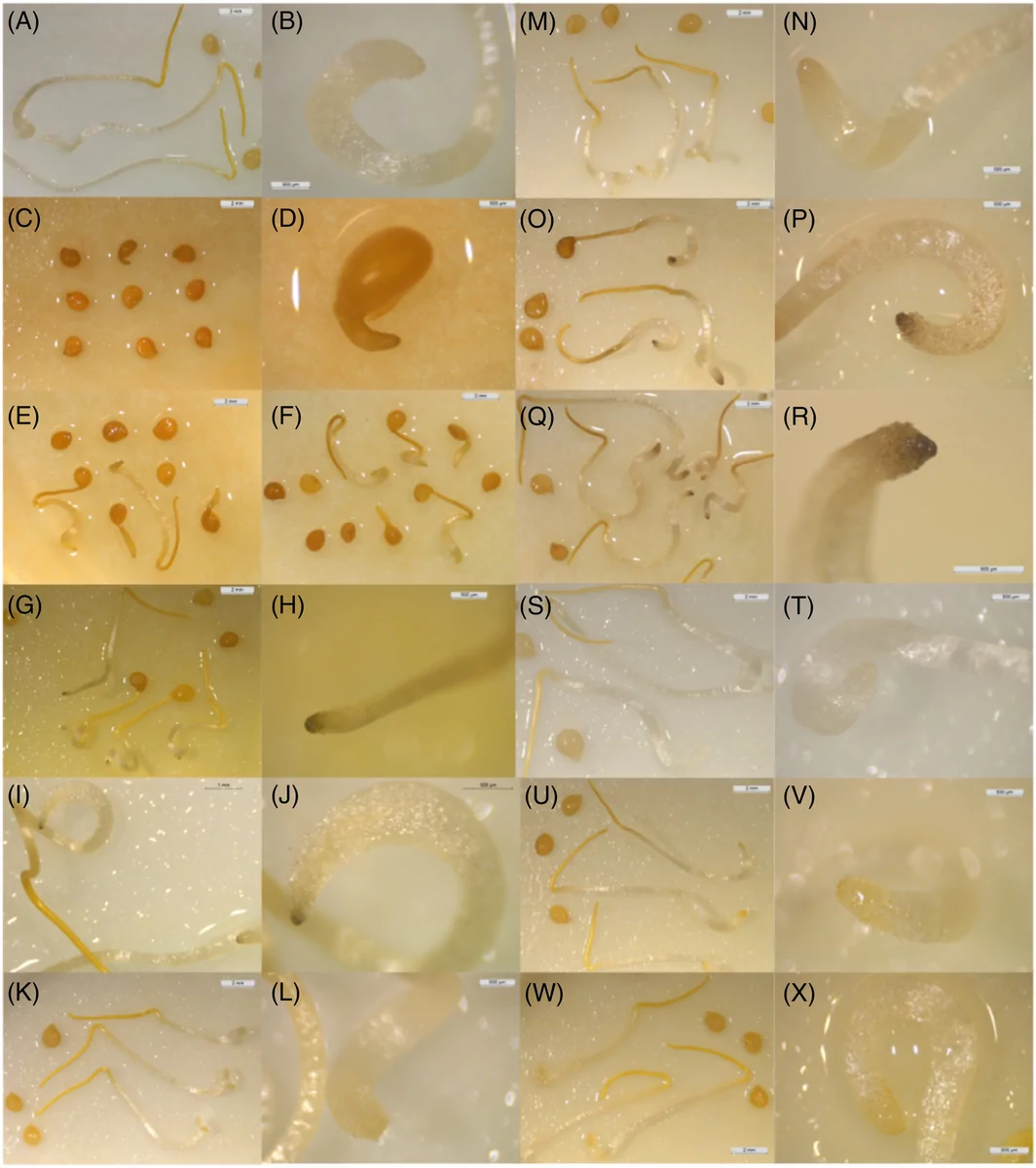
Representative photographs illustrating symptoms in *Cuscuta campestris* seedlings induced by treatments with control and compounds **1–3**, **7**, **9**, and **11–15**: Seedlings treated with control (A); detail of *C. campestris* root apice treated with control (B); effects in seedlings treated with APO (**1**) at 400 μM (C,D), at 200 μM (E) and at 100 μM (F); treated with product **2** at 400 μM (G,H); treated with product **3** at 400 μM (I,J); treated with product **7** at 400 μM (K,L); treated with product **9** at 400 μM (M,N); treated with product **11** at 400 μM (O,P) and at 200 μM (Q,R); treated with product **12** at 400 μM (S,T); treated with product **14** at 400 μM (U,V); treated with product **15** at 400 μM (W,X).

Besides APO (**1**) and the nitro derivative **11**, two additional phenozaxines induced measurable seedling necrosis: the dipyrido analogue (**2**) and the brominated pyridine derivative (**3**). Both produced necrosis over 80% at 400 μM, but their effects dropped sharply at lower doses and were no longer detected at 100 μM. Based on the estimated concentration–response trends on seedling necrosis, products **2** and **3** showed apparent IC_50_ values of 236.8 and 237.7 μM, respectively (in contrast, values below 100 μM were determined for the most bioactive products **1** and **11**).

Notably, the brominated dipyrido derivative **4** was inactive, suggesting that introduction of ring nitrogen atoms within the phenoxazinone framework can contribute to necrotic activity, although this effect is strongly substitution‐dependent. These trends observed among phenoxazinones **1–4** are consistent with SAR precedents on alternative structures, where introducing ring nitrogen atoms (pyridinyl or aza‐aromatics) or bromine substitution enhanced phytotoxic performance in weeds, with examples like phytotoxic pyridinyl‐benzothiazolones, 2‐((3‐pyridinyl‐2‐oxo‐2,3‐dihydrobenzo[d]thiazol‐6‐yl)oxy)propionates or bromoxynil derivatives against different types of weeds, as well as diverse natural and synthetic Br‐containing rubrolides targeting photosystem I.^37^, ^38^, ^39^, ^40^

The structural features discussed above for achieving phytotoxicity in *C. campestris* with phenoxazines **1–15** are summarized in Fig. 5.

**Figure 5:**
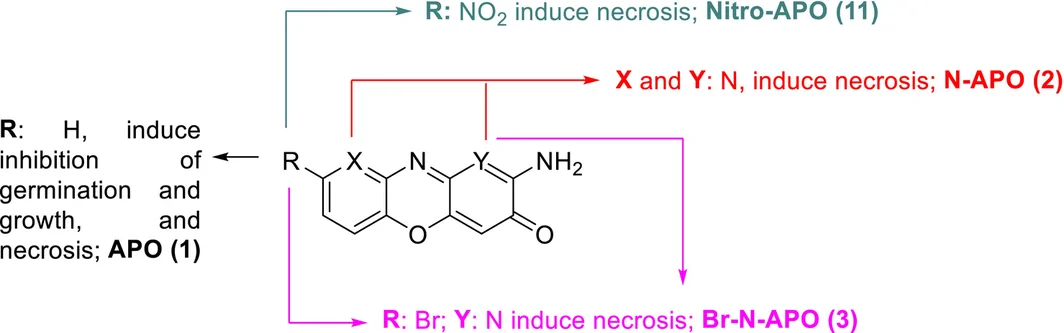
Summary of structure–activity relationships of the phytotoxicity of APO (**1**) and its bioactive derivatives **2, 3** and **11** in *Cuscuta campestris* early development.

Preliminary structure–activity relationships were further examined using calculated lipophilicity (through cLog *P* values), and TPSA, a 2D descriptor that estimates the surface contributed by polar atoms, thus relating to passive permeation and overall molecular polarity balance.^41^, ^42^ CLog *P* values ranged from 0.07 (**4**) to 3.08 (**8**), with the exception of product **2**, which showed a negative value (−0.81), all them in agreement with Tice's rule for post‐emergence herbicides.^43^, ^44^ Notably, the bioactive phenoxazinones **1**, **3** and **11** clustered at cLog *P* 0.92–1.14, yet the inactive molecules **9**, **12** and **14** displayed comparable values (1.29, 1.13 and 1.13, respectively), arguing against lipophilicity as a primary determinant of activity in *C. campestris*. Likewise, TPSA values spanned 69.12–114.94 Å^2^, and the active compounds (**1–3** and **11**) covered distinct positions within this interval (69.12, 94.90, 82.01 and 114.94 Å^2^ respectively), indicating that TPSA alone does not explain the activity differences.

The absence of a direct correlation with cLog *P* or TPSA suggests that the observed phytotoxicity is unlikely to be driven primarily by differences in transport or polarity, and is more consistent with scaffold‐dependent interactions in the target site. Nevertheless, considering both descriptors jointly reveals a qualitative trend, since the evaluation of compounds **1–15** provided results with molecules with similar TPSA, i.e., 69.12 Å^2^ and around 90 Å^2^ (Fig. 6). As depicted in Fig. 6, the more active phenoxazinones within these two TPSA values corresponded in both cases to the lower‐cLog *P* structures, i.e., APO (**1**) and the dipyrido analogue (**2**), respectively. This SAR conclusion suggests that a modest shift in phenoxazinone structures toward higher aqueous compatibility may favor bioexpression in *C. campestris*.

**Figure 6:**
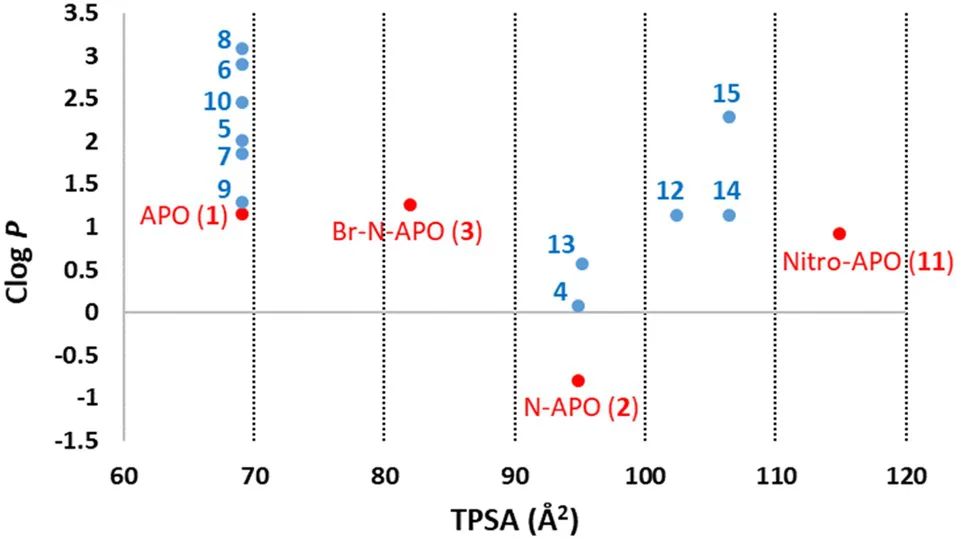
Graph of topological polar surface area (Å^2^) *versus* log *P* values of APO (**1**) and phenoxazines **2–15**. In red, compounds that showed any phytotoxic effect in *Cuscuta campestris* seedlings; in blue, inactive compounds.

Finally, it should be highlighted that control samples (2% DMSO in sterile distilled water) did not produce any observable phytotoxic effects, confirming that the bioactivity observed for APO (**1**) and derivatives **2**, **3**, and **11** during *C. campestris* early development is attributable to the tested compounds. It should be also emphasized that the mechanistic considerations previously discussed are hypothesis‐generating, since the present study was designed as a comparative SAR assessment of phenoxazinone effects on *C. campestris* early development. Notably, APO (**1**) has been reported to suppress *Arabidopsis thaliana* through inhibition of histone deacetylases HDA2 and HDA6,^19^ but whether this mechanism operates in *C. campestris*, or whether phenoxazinones trigger distinct parasite‐specific responses, remains unknown and will require targeted mechanistic studies. As recently reviewed, different types of aromatic and nitrogen‐containing natural products with phytotoxic effects in *C. campestris* have been discovered,^45^ which highlights the pertinence of further research on phenoxazinones as structurally distinctive allelopathy‐inspired scaffolds for developing new, targeted approaches against parasitic weeds.

## 4 CONCLUSION

APO (2‐amino‐3*H*‐phenoxazin‐3‐one) emerged as a robust reference phenoxazinone for the management of *C. campestris*, consistently impairing germination and early seedling growth, and inducing marked seedling necrosis in this parasitic weed. To the best of our knowledge, its bioactivity at the lowest tested concentrations represents the most potent effect reported to date on *C. campestris* early development. Structural modification of the scaffold generally did not improve the inhibition of germination or growth, reinforcing APO as a privileged allelochemical framework for these endpoints. In contrast, necrosis proved more tunable, since nitration enhanced the necrotic response at lower concentrations, while incorporation of ring nitrogens and bromine yielded additional necrosis‐active derivatives, albeit in a strongly substitution‐dependent manner. The lack of a direct relationship between activity and calculated cLog *P* or TPSA indicates that polarity or lipophilicity alone does not explain the bioactivity patterns, supporting a predominantly scaffold‐driven basis for the observed effects with a potential trend in favor of lower‐cLog P structures that share similar TPSA.

Taken together, these findings provide the first experimental support for phenoxazinones as lead models to target early developmental stages of dodder and, in general, a parasitic weed. The synthetic accessibility of phenoxazinones like APO at multigram scale makes this scaffold suitable for further research to define potency thresholds and applicative performance in higher scale assays for developing novel agrochemicals inspired in allelopathy, improving and reinforcing current management strategies. Elucidating the mode of action of phenoxazinones in *C. campestris* and parasitic plants will be an essential next step before placing their bioactivity in a broader context through comparison with reference herbicides.

## AUTHOR CONTRIBUTIONS

Conceptualization: JGZ. Methodology: MFA, JMGM and JGZ. Software: CDF, MFA and JGZ. Validation: MFA, JMG, RMV and JGZ. Formal analysis: MFA and JGZ. Investigation: CDF, MFA and JGZ. Resources: MFA, JMGM, RMV and FAM. Data curation: CDF, MFA and JGZ. Writing – original draft preparation: CDF, MFA and JGZ. Writing – review and editing: CDF and JGZ. Visualization: CDF and MFA. Supervision: JGZ. Project administration: MFA, JMGM, RMV and JGZ. Funding acquisition: MFA, JMGM, RMV, FAM and JGZ. All authors have read and agreed to the published version of the manuscript.

## CONFLICT OF INTEREST

The authors declare that they have no known competing financial interests or personal relationships that could have appeared to influence the work reported in this paper.

## Supporting information


**Figure S1–S15:** ^1^H and ^13^C NMR spectra of products 1–15 in deuterated dimethyl sulfoxide recorded at 500/125 MHz and their corresponding high‐resolution mass spectra.

## Data Availability

The data that support the findings of this study are available from the corresponding author upon reasonable request.
