# Supplementary material for: 2‐Amino‐3H ‐phenoxazin‐3‐one is a lead inhibitor of Cuscuta campestris early development: structure–activity relationships of o ‐aminophenol‐derived allelochemicals
Source: Pest Manag Sci. 2026 Jul 12;82(12):11234–43. doi: 10.1002/ps.71117 (PMC13756835; doi:10.1002/ps.71117)
Supplement: Supplementary file 1 [file PS-82-11234-s001.docx]

**Supporting Information**

**2-Amino-3*H*-phenoxazin-3-one is a lead inhibitor of *Cuscuta campestris* early development: Structure–activity relationships of *o*-aminophenol-derived allelochemicals**

Cristina Díaz-Franco^1,+^, Mónica Fernández-Aparicio^2,+^, José M. G. Molinillo^1^, Rosa M. Varela^1^, Francisco A. Macías^1^, and Jesús G. Zorrilla^1,*^

1 Allelopathy Group, Department of Organic Chemistry, Facultad de Ciencias, Institute of Biomolecules (INBIO), University of Cadiz, 11510 Puerto Real, Spain

2 Department of Crop Protection, Institute for Sustainable Agriculture (IAS), CSIC, Avenida Menéndez Pidal s/n, 14004 Córdoba, Spain

* Corresponding author. *E-mail address:* [jesus.zorrilla@uca.es](rewritten://92b430a5-436b-4b5f-92e9-3edf8f708ee8)

+ These authors contributed equally to this work

^1^H and ^13^C NMR spectra of products **1-15** in deuterated dimethyl sulfoxide recorded at 500/125 MHz and their corresponding high-resolution mass spectra

**S1.** 2-Amino-3*H*-phenoxazin-3-one (APO; questiomycin A; Phx-3; **1**) ...……………….. 2

**S2.** 2-Amino-3*H*-dipyrido[3,2-b:2′,3′-e][1,4]oxazin-3-one (**2**) ..……………………………. 3

**S3.** 2-Amino-8-bromo-3*H*-benzo[b]pyrido[2,3-e][1,4]oxazin-3-one (**3**) .…………………. 4

**S4.** 2-Amino-7-bromo-3*H*-dipyrido[3,2-b:2',3'-e][1,4]oxazin-3-one (**4**) ……………………5

**S5.** 2-Amino-8-bromo-3*H*-phenoxazin-3-one (**5**) …..……………………………………… 6

**S6.** 2-Amino-4,8-dibromo-1*H*-phenoxazin-1-one (**6**) ………………………...……………. 7

**S7.** 2-Amino-8-chloro-3*H*-phenoxazin-3-one (**7**) …………………..……………………… 8

**S8.** 2-Amino-8-chloro-4-iodo-3*H*-phenoxazin-3-one (**8**) ..………………………………… 9

**S9.** 2-Amino-8-fluoro-3*H*-phenoxazin-3-one (**9**) ………………………………………….. 10

**S10.** 2-Amino-8-fluoro-4-iodo-3*H*-phenoxazin-3-one (**10**) ……………………………… 11

**S11.** 2-Amino-8-nitro-3*H*-phenoxazin-3-one (**11**) ………………………………………... 12

**S12.** 2-Amino-3-oxo-3*H*-phenoxazine-8-carboxilyc acid (**12**) …………………………... 13

**S13.** 2-Amino-8-amino-3*H*-phenoxazin-3-one (**13**) ……………………………………… 14

**S14.** 2-Amino-3-oxo-3*H*-phenoxazine-7-carboxilyc acid (**14**) …………………………... 15

**S15.** 2-Amino-4-iodo-3-oxo-3*H*-phenoxazine-7-carboxilyc acid (**15**) ………………….. 16

**S1.** 2-Amino-3*H*-phenoxazin-3-one (APO; questiomycin A; Phx-3; **1**)

^1^H NMR spectrum of APO (**1**) in deuterated dimethyl sulfoxide.

^13^C NMR spectrum of APO (**1**) in deuterated dimethyl sulfoxide.

Negative-mode electrospray ionization mass spectrum (ESI–-MS) of APO (**1**) (calcd for C_12_H_8_N_2_O_2_, 211.0508).

**S2.** 2-Amino-3*H*-dipyrido[3,2-b:2′,3′-e][1,4]oxazin-3-one (**2**)

^1^H NMR spectrum of 2-amino-3*H*-dipyrido[3,2-b:2′,3′-e][1,4]oxazin-3-one (**2**) in deuterated dimethyl sulfoxide.

^13^C NMR spectrum of 2-amino-3*H*-dipyrido[3,2-b:2′,3′-e][1,4]oxazin-3-one (**2**) in deuterated dimethyl sulfoxide.

Negative-mode electrospray ionization mass spectrum (ESI–-MS) of 2-amino-3*H*-dipyrido[3,2-b:2′,3′-e][1,4]oxazin-3-one (**2**) (calcd. for C_10_H_6_N_4_O_2_, 211.01413).

**S3.** 2-Amino-8-bromo-3*H*-benzo[b]pyrido[2,3-e][1,4]oxazin-3-one (**3**)

^1^H NMR spectrum of 2-amino-8-bromo-3*H*-benzo[b]pyrido[2,3-e][1,4]oxazin-3-one (**3**) in deuterated dimethyl sulfoxide.

^13^C NMR spectrum of 2-amino-8-bromo-3*H*-benzo[b]pyrido[2,3-e][1,4]oxazin-3-one (**3**) in deuterated dimethyl sulfoxide.

Negative-mode electrospray ionization mass spectrum (ESI–-MS) of 2-amino-8-bromo-3*H*-benzo[b]pyrido[2,3-e][1,4]oxazin-3-one (**3**) (calcd. for C_11_H_6_BrN_3_O_2_, 289.9565).

**S4.** 2-Amino-7-bromo-3*H*-dipyrido[3,2-b:2',3'-e][1,4]oxazin-3-one (**4**)

^1^H NMR spectrum of 2-amino-7-bromo-3*H*-dipyrido[3,2-b:2',3'-e][1,4]oxazin-3-one (**4**) in deuterated dimethyl sulfoxide.

^13^C NMR spectrum of 2-Amino-7-bromo-3*H*-dipyrido[3,2-b:2',3'-e][1,4]oxazin-3-one (**4**) in deuterated dimethyl sulfoxide.

Negative-mode electrospray ionization mass spectrum (ESI–-MS) of 2-amino-7-bromo-3*H*-dipyrido[3,2-b:2',3'-e][1,4]oxazin-3-one (**4**) (calcd. for C_10_H_6_N_4_O_2_Br, 290.9518).

**S5.** 2-Amino-8-bromo-3*H*-phenoxazin-3-one (**5**)

^1^H NMR spectrum of 2-amino-8-bromo-3H-phenoxazin-3-one (**5**) in deuterated dimethyl sulfoxide.

^13^C NMR spectrum of 2-amino-8-bromo-3H-phenoxazin-3-one (**5**) in deuterated dimethyl sulfoxide.

Negative-mode electrospray ionization mass spectrum (ESI–-MS) of 2-amino-8-bromo-3H-phenoxazin-3-one (**5**) (calcd. for C_12_H_7_N_2_O_2_Br, 288.9613).

**S6.** 2-Amino-4,8-dibromo-1*H*-phenoxazin-1-one (**6**)

^1^H NMR spectrum of 2-amino-4,8-dibromo-1*H*-phenoxazin-1-one (**6**) in deuterated dimethyl sulfoxide.

^13^C NMR spectrum of 2-amino-4,8-dibromo-1*H*-phenoxazin-1-one (**6**) in deuterated dimethyl sulfoxide.

Negative-mode electrospray ionization mass spectrum (ESI–-MS) of 2-amino-4,8-dibromo-1*H*-phenoxazin-1-one (**6**) (calcd. for C_12_H_6_N_2_O_2_Br_2_, 366.8718).

**S7.** 2-Amino-8-chloro-3*H*-phenoxazin-3-one (**7**)

^1^H NMR spectrum of 2-amino-8-chloro-3*H*-phenoxazin-3-one (**7**) in deuterated dimethyl sulfoxide.

^13^C NMR spectrum of 2-amino-8-chloro-3*H*-phenoxazin-3-one (**7**) in deuterated dimethyl sulfoxide.

Positive-mode electrospray ionization mass spectrum (ESI–-MS) of 2-amino-8-chloro-3*H*-phenoxazin-3-one (**7**) (calcd. for C_12_H_8_N_2_O_2_Cl, 247.0274).

**S8.** 2-Amino-8-chloro-4-iodo-3*H*-phenoxazin-3-one (**8**)

^1^H NMR spectrum of 2-amino-8-chloro-4-iodo-3*H*-phenoxazin-3-one (**8**) in deuterated dimethyl sulfoxide.

^13^C NMR spectrum of 2-amino-8-chloro-4-iodo-3*H*-phenoxazin-3-one (**8**) in deuterated dimethyl sulfoxide.

Positive-mode electrospray ionization mass spectrum (ESI–-MS) of 2-amino-8-chloro-4-iodo-3*H*-phenoxazin-3-one (**8**) (calcd. for C_12_H_8_IN_2_O_2_Cl, 372.9241).

**S9.** 2-Amino-8-fluoro-3*H*-phenoxazin-3-one (**9**)

^1^H NMR spectrum of 2-amino-8-fluoro-3*H*-phenoxazin-3-one (**9**) in deuterated dimethyl sulfoxide.

^13^C NMR spectrum of 2-amino-8-fluoro-3*H*-phenoxazin-3-one (**9**) in deuterated dimethyl sulfoxide.

Negative-mode electrospray ionization mass spectrum (ESI–-MS) of 2-amino-8-fluoro-3*H*-phenoxazin-3-one (**9**) (calcd for C_12_H_6_FN_2_O_2_, 229.0413).

**S10.** 2-Amino-8-fluoro-4-iodo-3*H*-phenoxazin-3-one (**10**)

^1^H NMR spectrum of 2-amino-8-fluoro-4-iodo-3*H*-phenoxazin-3-one (**10**) in deuterated dimethyl sulfoxide.

^13^C NMR spectrum of 2-amino-8-fluoro-4-iodo-3*H*-phenoxazin-3-one (**10**) in deuterated dimethyl sulfoxide.

Negative-mode electrospray ionization mass spectrum (ESI–-MS) of 2-amino-8-fluoro-4-iodo-3*H*-phenoxazin-3-one (**10**) (calcd for C_12_H_5_F_1_I_1_N_2_O_2_, 354.9380).

**S11.** 2-Amino-8-nitro-3*H*-phenoxazin-3-one (**11**)

^1^H NMR spectrum of 2-amino-8-nitro-3*H*-phenoxazin-3-one (**11**) in deuterated dimethyl sulfoxide.

^13^C NMR spectrum of 2-amino-8-nitro-3*H*-phenoxazin-3-one (**11**) in deuterated dimethyl sulfoxide.

Positive-mode electrospray ionization mass spectrum (ESI–-MS) of 2-amino-8-nitro-3*H*-phenoxazin-3-one (**11**) (calcd for C_12_H_8_N_3_O_4_, 258.0515).

**S12.** 2-Amino-3-oxo-3*H*-phenoxazine-8-carboxilyc acid (**12**)

^1^H NMR spectrum of 2-amino-3-oxo-3*H*-phenoxazine-8-carboxilyc acid (**12**) in deuterated dimethyl sulfoxide.

^13^C NMR spectrum of 2-amino-3-oxo-3*H*-phenoxazine-8-carboxilyc acid (**12**) in deuterated dimethyl sulfoxide.

Negative-mode electrospray ionization mass spectrum (ESI–-MS) of 2-amino-3-oxo-3*H*-phenoxazine-8-carboxilyc acid (**12**) (calcd for C_13_H_7_N_2_O_4_, 255.0406).

**S13.** 2-Amino-8-amino-3*H*-phenoxazin-3-one (**13**)

^1^H NMR spectrum of 2-amino-8-amino-3*H*-phenoxazin-3-one (**13**) in deuterated dimethyl sulfoxide.

^13^C NMR spectrum of 2-amino-8-amino-3*H*-phenoxazin-3-one (**13**) in deuterated dimethyl sulfoxide.

Positive-mode electrospray ionization mass spectrum (ESI–-MS) of 2-amino-8-amino-3*H*-phenoxazin-3-one (**13**) (calcd for C_12_H_9_N_3_O_2_, 228.0773).

**S14.** 2-Amino-3-oxo-3*H*-phenoxazine-7-carboxilyc acid (**14**)

^1^H NMR spectrum of 2-amino-3-oxo-3*H*-phenoxazine-7-carboxilyc acid (**14**) in deuterated dimethyl sulfoxide.

^13^C NMR spectrum of 2-amino-3-oxo-3*H*-phenoxazine-7-carboxilyc acid (**14**) in deuterated dimethyl sulfoxide.

Negative-mode electrospray ionization mass spectrum (ESI–-MS) of 2-amino-3-oxo-3*H*-phenoxazine-7-carboxilyc acid (**14**) (calcd for C_13_H_9_N_2_O_4_, 255.0406).

**S15.** 2-Amino-4-iodo-3-oxo-3*H*-phenoxazine-7-carboxilyc acid (**15**)

^1^H NMR spectrum of 2-amino-4-iodo-3-oxo-3*H*-phenoxazine-7-carboxilyc acid (**15**) in deuterated dimethyl sulfoxide.

^13^C NMR spectrum of 2-amino-4-iodo-3-oxo-3*H*-phenoxazine-7-carboxilyc acid (**15**) in deuterated dimethyl sulfoxide.

Negative-mode electrospray ionization mass spectrum (ESI–-MS) of 2-amino-4-iodo-3-oxo-3*H*-phenoxazine-7-carboxilyc acid (**15**) (calcd for C_13_H_6_I_1_N_2_O_4_, 382.9529).
